# Atlanta Society of Medicine

**Published:** 1889-06

**Authors:** 


### ATLANTA SOCIETY OF MEDICINE.

MEETING April 15, 1889.

Hunter P. Cooper, M. D., President, in the chair.

### ANTISEPTICS IN OBSTETRICS.

Dr. Baird, addressing the Society on "Antiseptics in Obstetrics," said as follows: That he had selected this subject because of its general interest; not because he had anything new to offer, or opinion to set forth; but did so rather to find out what the several members thought upon this subject. It was natural to suppose that when antiseptics were first brought out that they would be applied to all branches of medicine, but he saw that there was a tendency to overdo, and to give too much credit to their power. Sometimes cases progressed just as rapidly and favorably without them as with them. The question was, when should they be used, before, during or after labor. In hospitals, it was, he believed, customary to use antiseptics before, during and after labor. In private practice he did not know of their use before the birth of the child, or during the birth of the child, but they were employed by some after labor. Again, what were the indications for their use, and what should be used? He held that antiseptics should not be employed indiscriminately, but only as circumstances demanded. He then read extracts from his paper on "the puerperal condition," published in *Reference Hand Book of Medicine*, relative to puerperal infection, etc. Said he read these extracts for the purpose of introducing puerperal fever, the most dreaded result of infection that the obstetrician had to meet. There was a great diversity of opinion as to the cause of this fearful disease, also as to the use of antiseptics in preventing it, and often its occurrence in relieving and shortening, or aborting the effects of the poison, whatever it may be. Dr. Baird then spoke of the use of uterine injection, when the temperature exceeded 100°, and also of the value of vaginal injections when there was a fetid condition of the lochial discharge. Said that he thought that the danger of uterine injection (injection into uterus) was really exaggerated, but that great care should be taken; always use a double canula; he used double canula and a Davidson's syringe. The solution he was in the habit of washing or injecting with was carbolic acid, one drachm to six ounces; this could be used with safety. The bichloride could also be used without danger to the patient—a solution of bichloride and muriate of ammonia in alcohol, as suggested to him by Dr. McRea, was an excellent one. Dr. Baird said that weak solutions were more frequently used now than strong ones, also that he, in his practice, used nothing after a normal labor, but if the lochial discharge was at all fetid used the vaginal injection himself, for one, two or three days, and on a rise of temperature he used uterine injections until the temperature fell to normal. After instrumental delivery he had seen cases get along all right without antiseptics; he had after, and during some instrumental deliveries, used antiseptics, and he had some cases in which he did not use antiseptics at all—the results were the same in both. So that he could not exactly tell where to draw the line. The negative cases proved more than the positive ones. He thought that the hands ought to be thoroughly scrubbed and disinfected before being placed in the vagina for any purpose, and that scrupulous cleanliness of the person of the patient and of the lying-in-room should be maintained at all times and under all circumstances.

Dr. Hardon said he was agreeably disappointed, for he had expected to hear a strong advocacy of antiseptics, and that he had, consequently, enjoyed Dr. Baird's remarks very much indeed. That so far as statistics were concerned they applied alone to hospital practice, had no bearing whatever upon private practice, and were no foundation to go upon. There were extremists on both sides, and there were middle men, and men who met the emergencies of each individual case, sometimes with antiseptics and sometimes without, no doubt; and the hospital records bear evidence of it that the death rate has been reduced (in the hospital) by the use of antiseptics. In private practice there are no statistics, and therefore we cannot tell much about it. Granting the value of antiseptics, we must take into consideration those great assistants—cleanliness and improved methods—strict cleanliness, isolation of cases, and not too frequent examination during labor. These all go to prevent infection, the praise is sometimes given to the antiseptics, when everything would have gone on just as well without them. In private practice normal labor needed no assistance; but Dr. Hardon said there was no such thing as normal labor, for the physician was sent for to assist the patient, and did so, thus the labor could not be strictly called normal. He said that it was remarked by some one, that if nature intended women to be treated antiseptically, she would have arranged the genital organs of woman so as to meet the emergency. Where antiseptics were employed very strict precautions should be taken, otherwise they were useless. All do not accept the microbic theory, therefore we are not compelled to hold to extreme methods. In normal labor antiseptics are superfluous, but he usually disinfected his hands, and if there was any tendency to fetid lochial discharge he used carbolic acid, 1 to 100, as vaginal injection, and if there was a rise of temperature to 102 degrees he recognized septic invasion, and used uterine injection every six hours until the temperature became fell to normal, and remained so. The solution used was the carbolic acid, 1 to 100. He was also in the habit of allowing all his patients, shortly after the birth of the child, to get up on a vessel and relieve their bowels and bladder; he thought, by this means, that the vagina and uterus were readily relieved of clots, and more thoroughly drained, and prevented septic absorption. He always used a double canula for uterine injection, placed the patient down on the bed, with her hips on the edge, used the fountain syringe; liked it best because more accustomed to it. In instrumental delivery, he always disinfected the instruments with a 1 to 40 per cent. solution of carbolic acid, which he has always found quite sufficiently strong. He also washed the vagina before and after with 1 to 100 of the carbolic acid solution. The conditions of abortion and miscarriage were different from natural labor. The placenta having in most instances to be taken away forcibly, there was a greater amount of surface, which gave rise to greater danger of absorption, and that the use of antiseptics in these cases was proper and right. He always curetted the uterus, and washed out the organ in every case of this kind, even if he was called after the ovum and placenta had been expelled. The solution he used was the carbolic acid one, 1 to 100, and had good results always.

Dr. Hagan said the question was one of difference between the antiseptic and the aseptic practice. If the aseptic theory was accepted and fully carried out, we have no need of employing antiseptics; but if asepsis was not thoroughly enforced, we certainly should use antiseptics to combat results that would obtain from the neglect of cleanliness. Said he did not think the solution as mentioned by Dr. Hardon, sufficiently strong to disinfect the instruments. He thought the antiseptic solution used by Gerster an excellent one; which was, two parts of salicylic acid, with twelve parts of boracic acid, to 1000. Dr. Hagan spoke also in this connection in favor of the use of a solution of boracic acid, following immediately the injection of the bichloride, as it had a tendency to prevent poisoning from the mercurial.

Dr. Kendrick said that he fully agreed with what had been said by Dr. Baird and Dr. Hardon. However, he had practiced for a number of years in the mountains of North Carolina, and had had many cases of obstetrics which he had treated without antiseptics of any kind whatsoever, and had had no bad results. Had used forceps many times without antiseptics, either before or after, and that every case terminated favorably. He never had a case of puerperal fever. He now accepted the advances of the day, and was impressed with the beneficial effects following the use of the several germicides.

Dr. Avary said that he had nothing to add to what had been already said; but simply that he was an advocate of the use of antiseptics, and that he had enjoyed Dr. Baird's address very much indeed.

Dr. McRae said that he was in accord with Dr. Baird's views, but that Dr. Baird did not go far enough. That because there was no trouble, was no reason why we should not use all precautions; there was a great responsibility in every case, and all alike needed vigilant care and nursing, and every precaution taken. Said he did not think 1 to 40 solution of carbolic acid was strong enough to effect the purpose. That he used 1 to 1000 bichloride solution, both to wash his hands in and to disinfect the instrument; this solution had no bad effect upon the hands. That since he had begun the use of antiseptics he had no trouble. His plan was to leave the instruments in the bichloride solution for a half hour; wash all the parts with the same solution, and his hands, before touching the patient. Always used the antiseptic pad. There was no comparison to be made between the use of antiseptics in general surgery and in obstetrics.

Dr. Joye said that he thought the use of antiseptics was not to be objected to, and that he agreed fully with the excellent and conservative remarks of Dr. Baird, also with what Dr. Hardon had said on the subject. His opinion was, that when antisepsis was adopted that it should be carried out thoroughly and faithfully in its every detail. That in many instances, the letter of the law was not strictly adhered to. That soaking the instruments, etc., in a weak solution, for but a short time, did not appear to meet the indications. That all instruments to be used should be placed in a *pan with lamp beneath* (like the old fashioned chopping dish), so that the water in which the instruments lie can be kept constantly boiling. He claimed that all instruments, for the purpose of washing out the uterus or vagina, should have *glass nozzles*, *long enough*, so as to prevent the rubber tubing from coming in contact with any portion of the uterine or vaginal tissue. He said also, that he believed many women to be infected by the use of the gutta-percha nozzles, of the popular syringes; that if these nozzles were scraped, and the scrapings put under the microscope, that one would be surprised at what they saw.

That the essence of antisepsis was to have air, patient, instruments, surgeons, assistants, room and everything in it under the influence of the antiseptic all the time, and that that could not be done except by some such chamber as suggested by David Prince, M. D., a description of which is given in Vol. II, Reports Ninth International Medical Congress. Dr. Joye also remarked that he did not believe that the mortality attending obstetrical cases in the country and private city practice to have been greater before than since the adoption of antiseptics in the management of the puerperal woman.

Dr. Cooper said that so far as statistics were concerned, they had no bearing upon the question, because they were all taken from hospital readers. That the practitioner should shape his treatment according to the necessities of each individual case. That there was a great difference of opinion as to the use of and the value of antiseptics. That some physicians did believe in the efficacy of antiseptics, and others did not. That he thought in healthy localities there may be no need of them, and in others there was an imperative demand for their use. In the Vienna General Hospital, where he had the pleasure of being an interne in Carl Browne department, the technique of antisepsis was very thorough. The patients came a week or so before confinement was expected, and on admission were subjected to a hot soap bath, to remove the outer layer; in addition to this, a vaginal douche of hot water every two days preceding labor, and after labor an antiseptic vaginal injection. That the rules governing the students, or internes, were very strict. They were required to wash and scrub their hands, then wash them in a 1 to 1000 solution of bichloride; then in a solution of permanganate of potash; this had to be done every time they examined a patient. Said he had used the antiseptic uterine injection but twice, and then only when the temperature was above normal; in the cases in which he employed it the temperature was 105 and 104½ respectively. That it was surprising how rapidly the temperature fell after the use of antiseptic uterine injection, and that he had no hesitancy in saying that the injection was the potent agent in relieving all the untoward symptoms. He said the double metallic catheter was easily cleansed and was the proper instrument for uterine injection. He used this, together with the fountain syringe. That decomposed material was as apt to form on, and behind the labia as anywhere else, and that this, if cleanliness was not enforced, was oftentimes carried up into the vagina, and was the cause of septic infection. Said that he had been much pleased with the views as expressed by Dr. Baird, and was glad to say that they met with his earnest support and approval.

Dr. Baird said, in closing the discussion, that he was gratified in having nearly all who had spoken on the subject agree with him. We did not know the cause of the infection, and consequently could not follow any definite method of treatment—that he would not be carried away with the idea that carbolic acid and bichloride would kill anything; that in years to come, it may be demonstrated that neither of these drugs was at all effective as antiseptics, or germicides. That he did not find moving the patient to edge of bed necessary when using the uterine injection; with regard to abortion, he never curetted the uterus; that in many cases it was difficult to get into the womb, and he thought curetting would increase the danger of the condition. His treatment, if there was hemorrhage, was to tampon the vagina, placing next to the *os uteri* a piece of cotton well dusted with iodoform, then packed around this; allowed this dressing or tampon to remain twelve hours, then removed all, and washed out the vagina with carbolized water, re-applied iodoform and cotton to *os*, and repacked vagina as before, and continued this as long as it was necessary, and always had had good results. Dr. Baird mentioned a puerperal case, where he had given veratum viride for the relief of fever and other symptoms, and the woman had a collapse; after being brought out of the collapsed state, she had no more fever or further trouble. Said if he had used antiseptic uterine injection in this case, that all the credit would have been given to the antiseptic.

Dr. Baird closed by thanking the members for their kind attention to his remarks:

